# Geographical variation and factors associated with gastric cancer in Manitoba

**DOI:** 10.1371/journal.pone.0253650

**Published:** 2021-07-09

**Authors:** Oluwagbenga Fakanye, Harminder Singh, Danielle Desautels, Mahmoud Torabi

**Affiliations:** 1 Department of Community Health Sciences, University of Manitoba, Manitoba, Canada; 2 Department of Internal Medicine, University of Manitoba, Manitoba, Canada; 3 Research Institute in Oncology and Hematology, CancerCare Manitoba, University of Manitoba; Institute of Science, Banaras Hindu University, INDIA

## Abstract

**Objectives:**

We investigated the spatial disparities and factors associated with gastric cancer (GC) Incidence in Manitoba.

**Methods:**

We combined information from Manitoba Cancer registry and Census data to obtain an age-sex adjusted relative risk (IRR) of GC incidence. We geocoded the IRR to the 96 regional health authority districts (RHADs) using the postal code conversion file (PCCF). Bayesian spatial and spatio-temporal Poisson regression models were used for the analysis.

**Results:**

Adjusting for the effect of socio-economic score index (SESI), Indigenous, and immigrant population, 25 districts with high overall GC risk were identified. One unit increase in SESI was associated with reduced risk of cardia GC (CGC) by 14% (IRR = 0.859; 95% CI: 0.780–0.947) and the risk of non-cardia GC (NCGC) by approximately 10% (IRR = 0.898; 95% CI: 0.812–0.995); 1% increase in regional Indigenous population proportion reduced the risk of CGC by 1.4% (IRR = 0.986; 95% CI: 0.978–0.994). In the analysis stratified by sex, one unit increase in SESI reduced the risk of CGC among women by 26.2% (IRR = 0.738; 95% CI: 0.618–0.879), and a 1% increase in Indigenous population proportion reduced the risk of CGC among women by 1.9% (IRR = 0.981; 95% CI: 0.966–0.996).

**Conclusion:**

Our results support a significant association between SESI and NCGC. We report regional variation of GC IRR and a varying temporal pattern across the RHADs. These results could be used to prioritize interventions for regions with high and progressive risk of GC.

## Introduction

One of the cancers that has consistently contributed to the global burden of cancer is Gastric Cancer (GC). It is the fifth most diagnosed cancer worldwide, which accounts for 6.1% of all cancer cases for both sexes. It stands as the fourth most diagnosed cancer in men accounting for 7.8% of all cancers affecting men globally and the seventh most diagnosed cancer in women accounting for 4.3% of all cancers affecting women globally [1].

Almost half (43.5%) of all the GC cases are diagnosed at an advanced stage (stage IV), where cancer has metastasized to other parts of the body [2]. Therefore, only one third of all diagnosed GC cases live beyond five years, which is one of the lowest cancer survival rates [3]. In some parts of the world, researchers have studied the geographical and temporal variation of GC with the sole purpose of understanding its propagation [4–7]. However, there is very limited information about the geographical variation in Canada. The purpose of this study was to identify hot-spot districts and associated area-level risk factors of GC, across the 96 Regional Health Authority Districts (RHADs) in Manitoba using a twenty-five year (1992–2016) Canadian Cancer Registry (CCR) dataset. Specifically, this study investigated the geographical variation of GC incidence in Manitoba, explored factors influencing the geographic difference of GC incidence in Manitoba, and also investigated the geographic variation of GC incidence over time in Manitoba.

## Materials and method

### Study area

**Fig 1:**
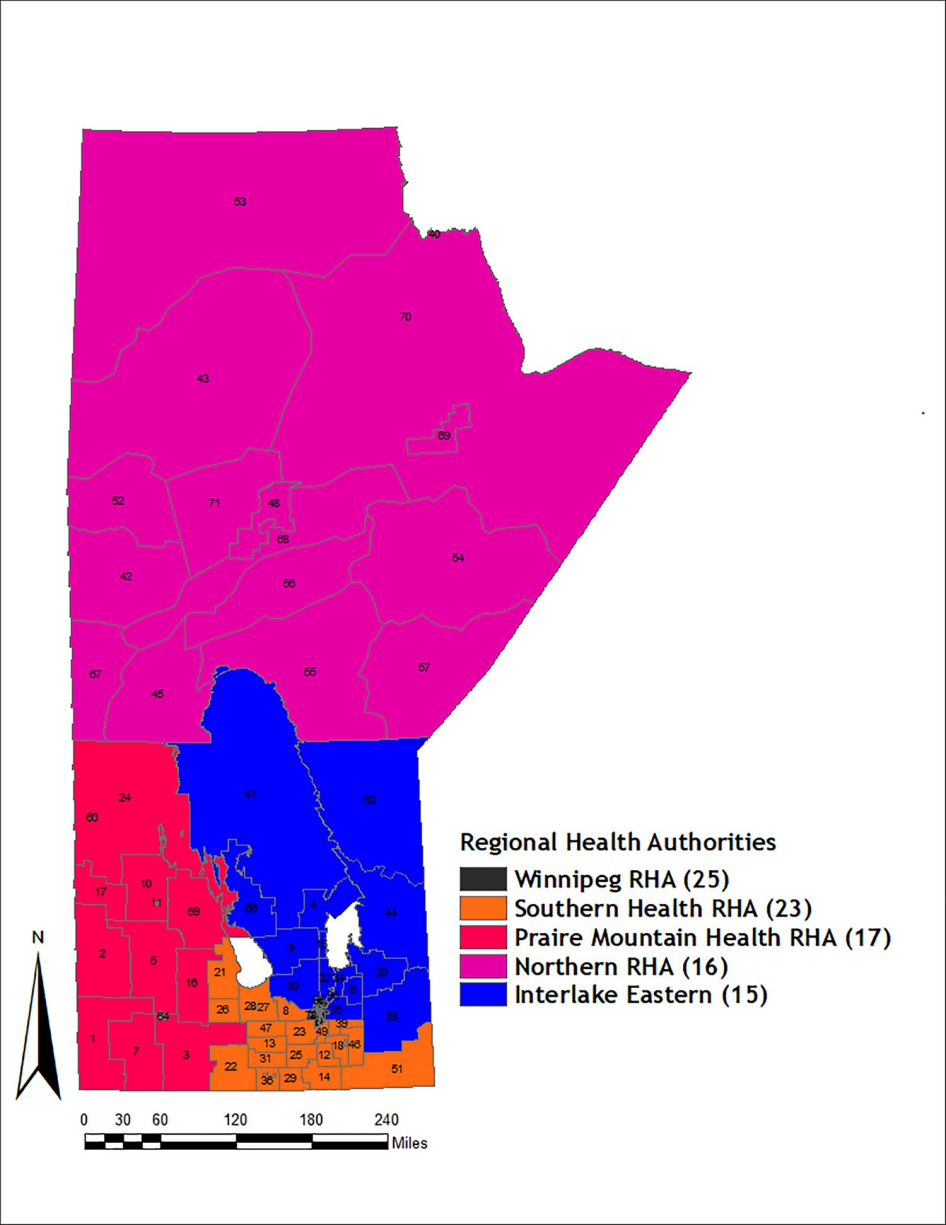
Map of five Regional Health Authorities (RHA) and the corresponding Regional Health Authority Districts in Manitoba. Number of districts for each RHA is shown in parenthesis in the legend. *Note: The numbers on the map and subsequent ones in this study represents area identification tag.

Manitoba, the study region, has an approximate population of 1,278,365 based on the 2016 population census. 631,400 (49.39%) identified as male, while 646,965 (50.61%) identified as female. Winnipeg is the capital of the province, with more than half (55.96%) of the entire Manitoba population. It is a diversified multi-cultural ethnic province with a higher proportion of Indigenous peoples than many other Canadian provinces [8]. The province has five regional health authorities (RHAs) created from the initial eleven RHAs in 2012. These RHAs are tasked with overseeing health services, both acute and community-based care. The RHAs are further sub-divided into districts (RHADs), [9]. The RHADs (Fig 1), were adopted in this study as the area-level aggregate point. At the time of this study, the province was divided into 96 RHADs, which were used as the analysis unit in this study as used in other similar geographical studies [10]. ArcGIS, version 10.2.2 [11], was used to create choropleth maps displayed in subsequent sections. All maps presented in this study are created from the shapefiles which form part of the dataset used in the analysis. It does not require a copyright permission.

### Data

Using the universal disease classification code, as defined by the international classification of disease for oncology third Edition (ICD-O3) [12], we extracted all cases of GC (C160 –C169) in Manitoba from 1992 to 2016 from the Canadian Cancer Registry (CCR) (Table 1). Prior to 1992, there was no unified dynamic database for cancers in Canada [13].

**Table 1:** Description of all GC topographies using ICD-O3.

ICDO-O3 code	Topography sub-site
C160	Cardia
C161	Fundus
C162	Body
C163	Antrium
C164	Pyloric
C165	Lesser curvature of the stomach
C166	Greater Curvature of stomach
C168	Overlapping lesion of the stomach
C169	Stomach unspecified

We also obtained demographic data (age and sex) from the CCR data. Upon the satisfaction of the ethical condition requirement by the Research Data Centre (RDC) and a course completion on research ethics by the Tri-Council Policy statement Ethical Conduct (TCPS2: CORE), we were granted access to the CCR dataset from the Research Data Centre (RDC) repository located at the University of Manitoba campuses (Bannatyne and Fort-Gary). All data extracted from the CCR used in this study were fully anonymized before access to avoid bridging of confidentiality. We created an age-sex standardized incidence risk (ASSIR). We obtained information for average socioeconomic status in RHADs, proportion of individuals of Indigenous and immigrant population in each RHAD from the 2016 Census datasets. The 2016 Census data was used based on the assumption that the population of Manitoba within the studied period has been relatively steady (1.1 million– 1.2 million).

#### Socioeconomic status score index

Several studies have linked Socioeconomic status (SES) with the etiology of GC [14–16], which partly contributed to the need to adjust for it in this study. The socioeconomic status score index (SESI) used in this study was computed via a linear combination of income, employment and education using factor analysis (FA); a method that has been widely used in SESI computation [17–19]. We adopted the FA algorithm implemented by [20], where SESI value ranged from 0.643397 to 9.4163. The lowest score indicates poor socio-economic status while the highest score indicates a good socio-economic status (high standard of living).

#### Immigrant population

Considering the diversity in Manitoba population, another factor of interest is the distribution of immigrant across the province. This factor is considered important in this study as our interest is on geographical variation of GC which could be linked to corresponding geographical variation in the distribution of immigrants across the province. There have been studies that looked into the association between GC and immigration in different countries. However, the result has been inconsistent [21–23]. The measure of immigrant population variable used in this study was the districts’ percentage population of individuals residing in Manitoba during the study period who identified themselves as an immigrant after 1965 (according to the definition of 2016 Canadian Census).

#### Indigenous population

Another essential factor of interest that has extensively been researched in the epidemiological study of GC in Canada is the Indigenous population [6, 24, 25]. Despite the extensive study of this factor, little information is available about its impact on geographical variation of GC in Manitoba. The measure of Indigenous population variable, as used here, represents the districts’ percentage of Manitoba population who identified as First Nation, Metis or Inuit.

### Statistical analysis

Information extracted from the CCR and the 2016 Census data were merged using the postal code conversion file (PCCF) which provides a correspondence between the Canadian six-alphanumeric postal code and Statistics Canada’s standard geographic area. We standardized the GC incidence by age and sex since our primary focus did not include these two variables. This was also done to eliminate the possible impact of age and sex on the parameter estimates which may lead to spurious estimates. GC cases were stratified into cardia and non-cardia GC based on topographical sub-site, and into 5-year interval (1992–1996, 1997–2001, 2002–2006, 2007–2011, 2012–2016) to examine the risk of GC over time; note that we split our data into 5-year category to avoid sparsity in our dataset. A Moran’s I statistic was used as a confirmatory test to assess the spatial dependency of GC, as a justification for using spatial regression model. Bayesian spatial and spatio-temporal Poisson regression models are mathematically defined later in Eqs (1.2) and (1.4) respectively were used to address the research objectives [26–30].

#### Bayesian regression model

Let *y*_*ijk*_;*i* = 1,…,96;*j* = 1,2;*k* = 1,..,12 represents the GC count in RHAD *i*, sex group *j* and age group *k* = *less than* 35, 35−39, 40−44,…,85+; *y*_*ik*_ is the GC count in sex *j* and age group *k*; ***y***_***i***_; I = 1, …, 96 is the GC count in RHAD *i*; *n*_*ijk*_ is the population of people in RHAD *i* belonging to sex group *j* and age group *k*; *n*_*jk*_ is the population of people in sex *j* and age group *k*; *x*_*i*_ is the covariates in RHAD *i*, (SESI, the proportion of Indigenous, the proportion of immigrants), and assuming the total number of cases of GC in each RHAD follows a Poisson distribution, i.e. *Y*_*i*_~*Poisson*(*λ*_*i*_*E*_*i*_), where *λ*_*i*_ is relative risk and *E*_*i*_ is the expected number of GC count in RHAD *i* defined in Eqs (1.0) and (1.1),

$$Y_{i}=\sum_{j=1}^{2}\sum_{k=1}^{12}y_{ijk}$$ (1.0)

$$E_{i}=\sum_{j=1}^{2}\sum_{k=1}^{12}\frac{y_{jk}}{n_{jk}}\times n_{ijk}$$ (1.1)

The spatial regression model used in this study is mathematically defined as

$$log(\lambda_{i})=\alpha +\beta x_{i}+u_{i}+\eta_{i}$$ (1.2)

where *α* and *β* are mean ratio (intercept) and regression coefficients, and *η*_*i*_ and *u*_*i*_ are the spatially correlated and uncorrelated random effect respectively [26–28]. The *u*_*i*_, $u_{i}∼N(0,\sigma_{u}^{2})$, is the unstructured heterogeneity, “noise,” which denotes the variation unaccounted for in this study. The *η*_*i*_ is the structured heterogeneity described by a Gaussian conditional autoregressive (CAR) distribution which is defined as

$$\eta_{i}|\eta_{j,\;i\neq j},\tau_{\eta }∼N\left(\frac{1}{N(i)}\sum_{j∼i}^{n}w_{ij}\eta_{j},\;\frac{\sigma_{\eta }^{2}}{N(i)}\right)$$ (1.3)

The N(i),i=1,…,96, is the set of neighbors(s) to a specific RHAD which can be defined in different ways such as proximity or boundary sharing between regions [31]. The neighborhood as used in this study is defined as two regions sharing boundaries, and *w*_*ij*_ represent the relationship between any two regions (*i*,*j*), which is defined as

$$w_{ij}=\left\{ \begin{array}{l} 1,\;i\;and\;j\;are\;adjacents\\ 0,\;otherwise \end{array}\right.$$

and $\sigma_{\eta }^{2}$ is the spatial dispersion of region *i*. In other to account for the temporal effect in the spatial model, Eq (1.2) was extended as shown in Eq (1.4) below:

$$log(\lambda_{it})=\alpha +\beta x_{i}+u_{i}+\eta_{i}+\gamma_{t}+\varphi_{t}$$ (1.4)

where the additional two components (*γ*_*t*_, *φ*_*t*_) represent the unstructured and structured temporal effects. The structured temporal effect, *φ*_*t*_, is modeled by imposing a random walk of order one, RW (1), a distribution which is defined as a step function given as

$$\varphi_{t}|\varphi_{-t}∼\left\{ \begin{array}{c} Normal\;(\varphi_{t+1},\sigma_{\varphi }^{2}),\;for\;t=1\\ Normal\;(\frac{\varphi_{t-1}+\varphi_{t+1}}{2},\frac{\sigma_{\varphi }^{2}}{2}),\;for\;t=2,\ldots ,T-1\\ Normal\;(\varphi_{t-1},\sigma_{\varphi }^{2}),\;for\;t=T \end{array}\right.$$

and an exchangeable prior $\gamma_{t}∼(0,\sigma_{\gamma }^{2})$ is imposed on the unstructured temporal effect. The number of parameters and hyperparameters to be estimated are *θ* = (*α*,*β*) and $\psi =(\sigma_{u}^{2},\sigma_{\gamma }^{2},\sigma_{\varphi }^{2},\sigma_{\eta }^{2})$ respectively. The model fitting was implemented using Integrated Nested Laplace Approximation (INLA) in R programming language. We imposed different minimally informative prior on the log of the structured effect precision to examine the effect of prior on the parameter estimates. In summary, we imposed an intrinsic conditional autoregressive (ICAR) to account for the spatial dependency in both spatial and spatio-temporal model, using a minimally informative uniform prior distribution for the intercept *α*~*U*(−∞, +∞), a normal prior distribution for the slopes *β*~*N*(0, 1000), and a log Gamma prior for both spatially structured and unstructured precision log *τ*~log *Gamma* (0.1, 0.01). We present the incidence risk ratios (IRR) and the corresponding 95% credible interval for the model parameters.

## Results and discussion

### Results

**Fig 2:**
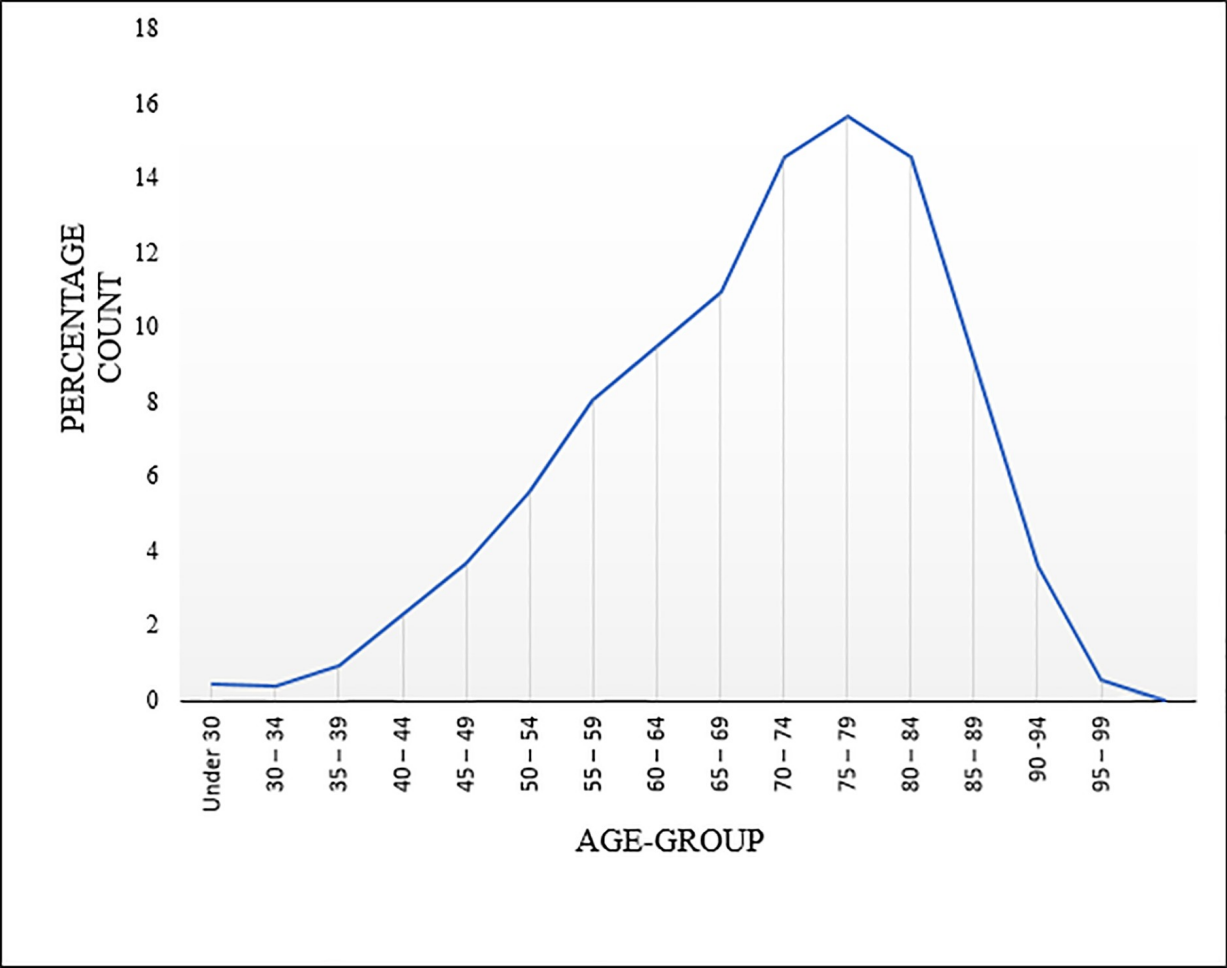
GC distribution by age group.

A total of 3,172 cases of GC were diagnosed between 1992 and 2016 in Manitoba. Approximately 31% of the GC cases were cardia gastric cancer (CGC), 49% were non-cardia gastric cancer (NCGC) and 20% were unspecified. The highest number of GC (approximately 16%) occurred in the group 75–79 as shown in Fig 2.

**Fig 3:**
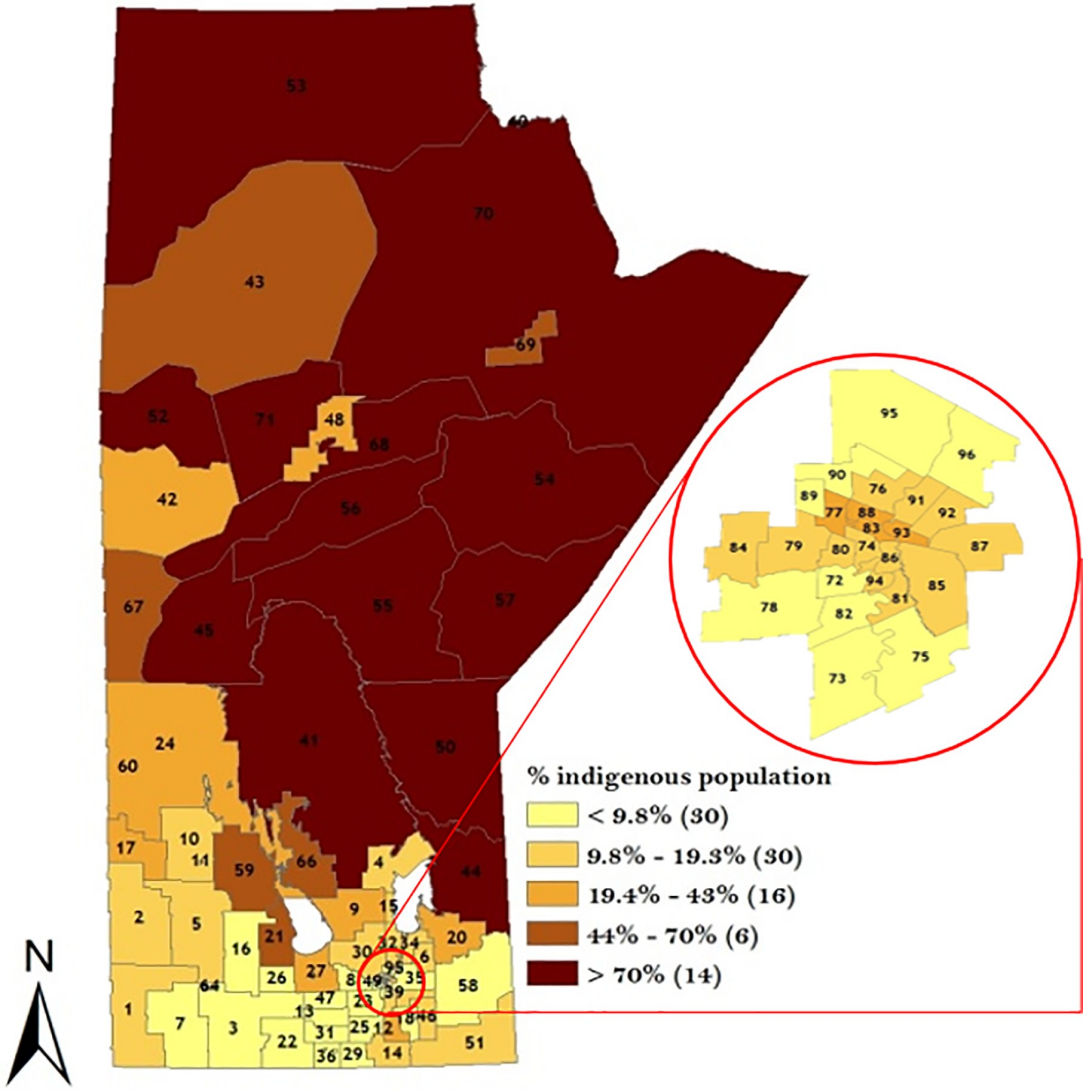
Geographical distribution of Indigenous population across the 96 RHADs based on 2016 Canadian Census data. Numbers in the map are the district labels from 1 to 96. Values in parenthesis represents the counts in each distribution category.

**Fig 4:**
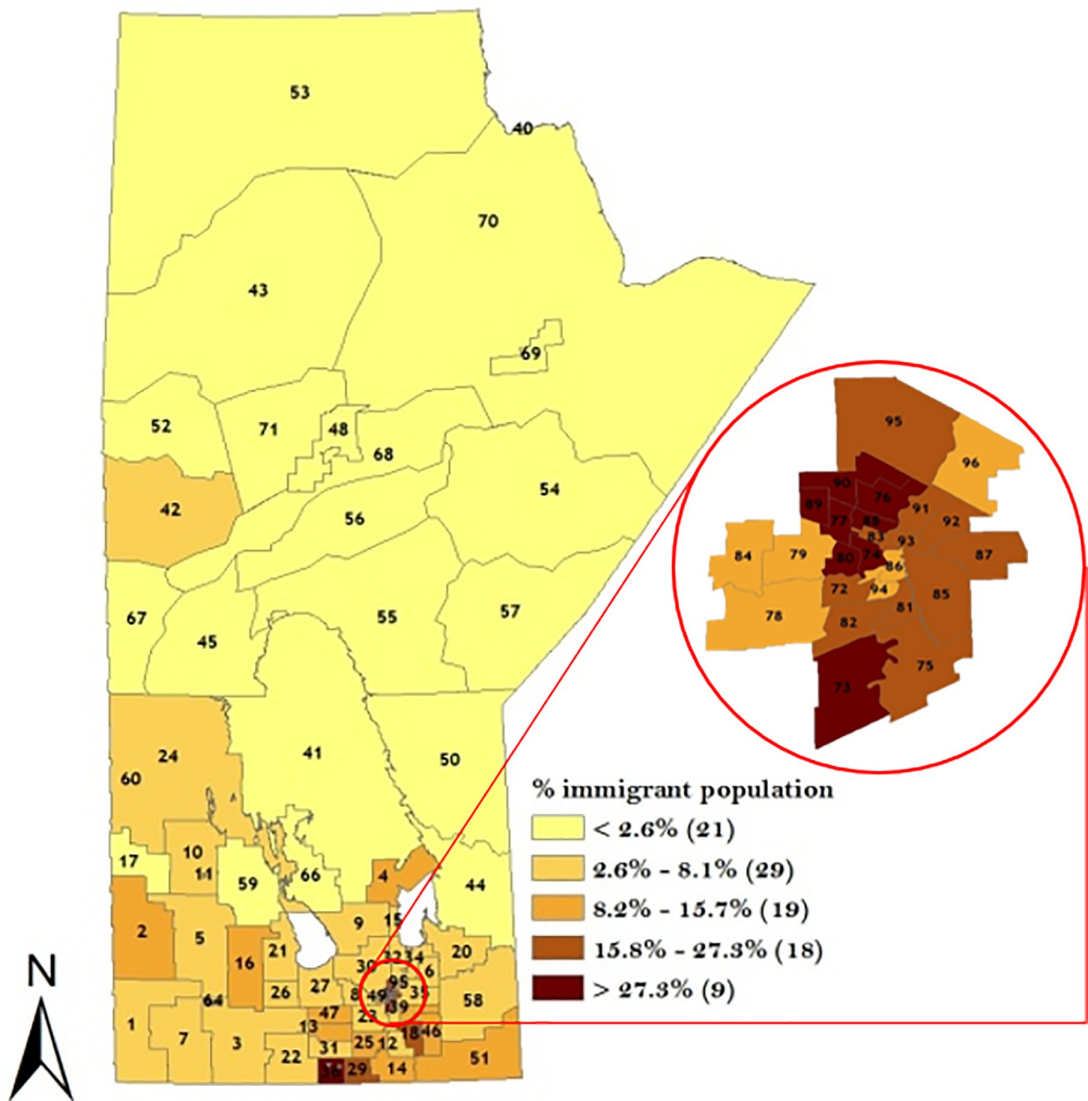
Geographical distribution of immigrant population across the 96 RHADs based on 2016 Canadian Census data. Numbers in the map are the district labels from 1 to 96. Values in parenthesis represents the counts in each distribution category.

**Fig 5:**
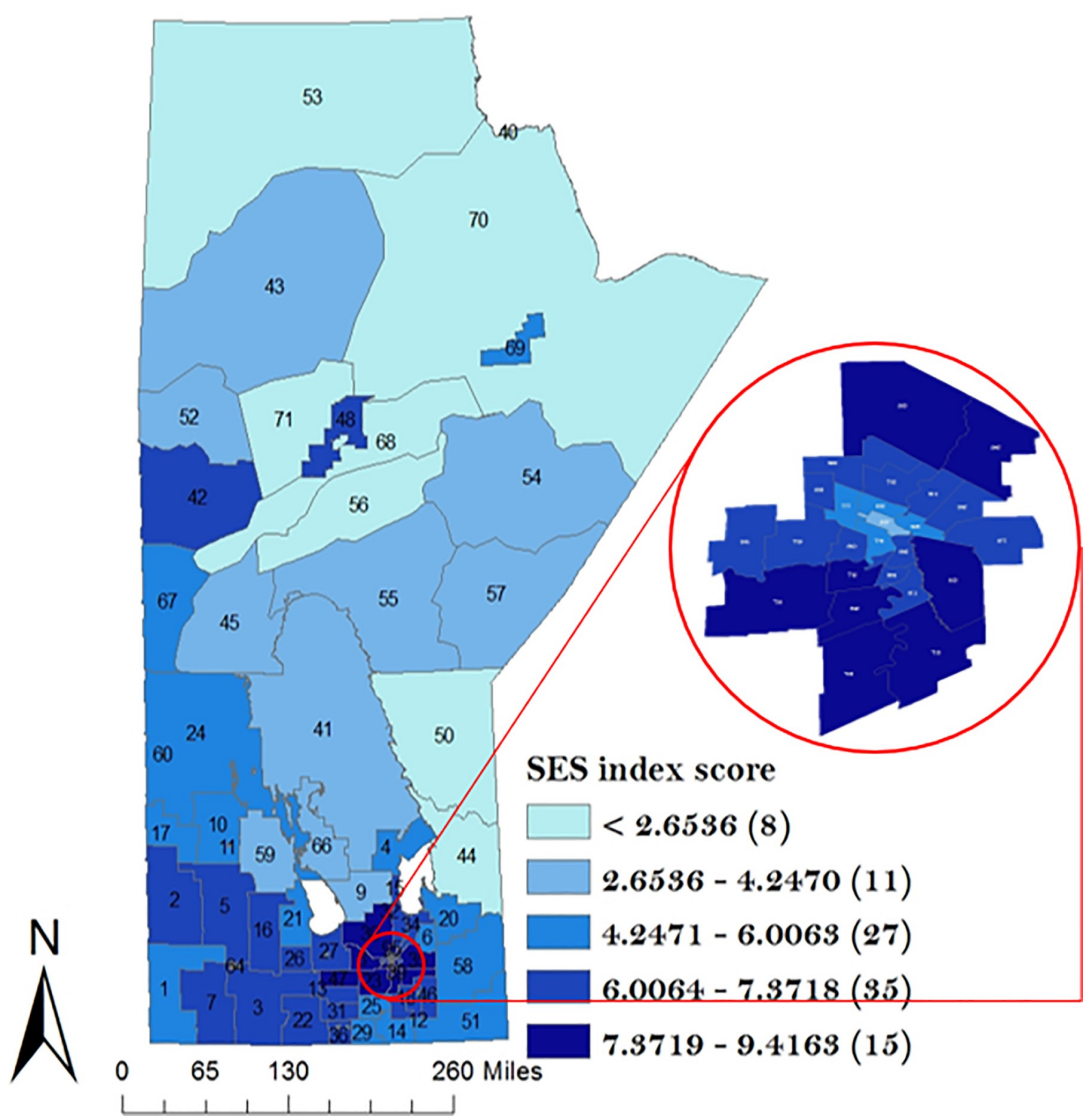
Geographical distribution of socio-economic status score index across the 96 RHADs based on 2016 Canadian Census data. Numbers in the map are the district labels from 1 to 96. Values in parenthesis represents the counts in each distribution category.

A descriptive analysis of the potential risk factors is presented in Figs 3–5. The proportion of Indigenous people was highest in the north-western part of the Manitoba (Fig 3) while the proportion of immigrants was highest in the Winnipeg Regional Health Authority (WRHA) (Fig 4). Also, the SESI revealed that majority of the districts in the northern Manitoba had the lowest SESI, which translates to having a low socio-economic status, while WRHA and the majority of the southern part of Manitoba had a high SESI (Fig 5).

**Fig 6:**
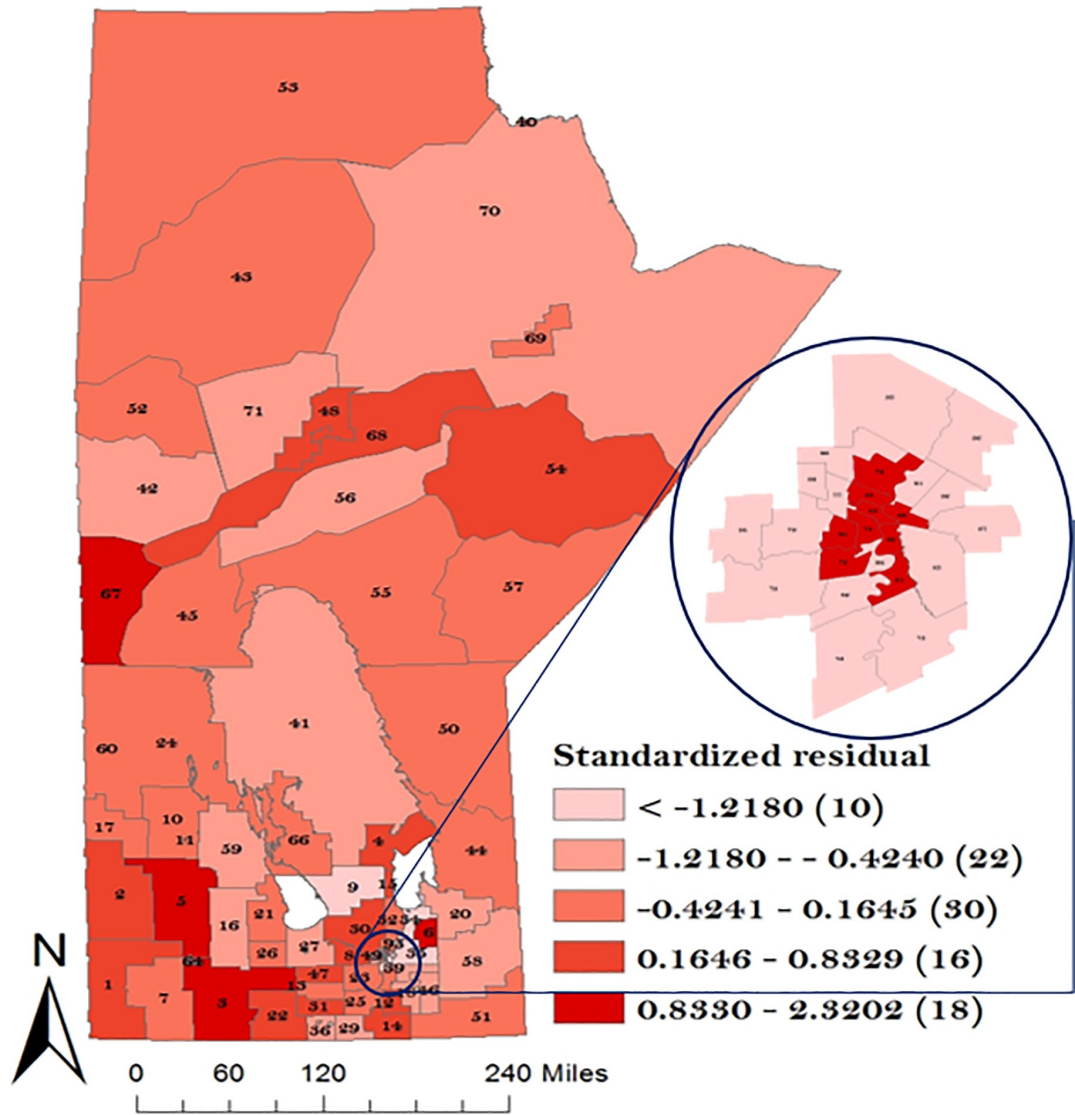
Map of unexplained variation in overall GC incidence risk ratio. Numbers in the map are the district labels from 1 to 96. Number of districts in each risk category is shown in parenthesis in the legend.

We first fitted a multivariable Poisson regression model to examine the spatial dependency of overall GC IRR. This was done by examining the unexplained variation in GC IRR as shown in Fig 6. The result revealed the presence of geographical dependency of GC which is evident based on the variation in color gradient in the map. Region(s) with lighter color indicates low GC IRR, while darker color indicate regions with high GC IRR as used in this study for all maps.

This result was followed by a confirmatory test using Moran’s I statistics defined as

$$I=\frac{n}{S_{0}}\left(\frac{\sum_{i=1}^{n}\sum_{j=1}^{n}w_{ij}(y_{i}-\bar{y})(y_{j}-\bar{y})}{\sum_{i=1}^{n}{\left(y_{i}-\bar{y}\right)}^{2}}\right)$$ (1.5)

where *y*_*i*_ represents the count of GC cases in region *I*; $\bar{y}$ represents the average count of GC cases; *w*_*ij*_ represents the distance weight matrix obtained assuming a neighborhood structure where regions share a direct boundary with one another; *n* is the number of regions (n = 96); $S_{0}=\sum_{i=1}^{n}\sum_{j=1}^{n}w_{ij}$ is the aggregate weight. The Moran’s I statistic confirmed the existence of spatial dependency (clusters) (0.1563; p-value = 0.007). Tables 2 and 3 present the effects of the risk factors on our dataset partitioned by sex and topographical sub-sites respectively.

**Table 2:** Incidence Risk Ratio (IRR) and 95% credible interval for overall, male and female GC dataset using spatial Poisson regression model.

Parameter	Overall population		Male population		Female population	
	IRR	95% Credible Interval (Lower, Upper)	IRR	95% Credible Interval (Lower, Upper)	IRR	95% Credible Interval (Lower, Upper)
**SESI**	0.921	(0.836, 1.013)	0.916	(0.833, 1.008)	0.906	(0.814, 1.010)
**Immigrant**	1.003	(0.992, 1.014)	1.004	(0.993, 1.014)	1.003	(0.992, 1.014)
**Indigenous**	1.001	(0.994, 1.008)	1.000	(0.992, 1.007)	1.004	(0.961, 1.012)

**Table 3:** Incidence Risk Ratio (IRR) and 95% credible interval for cardia and non-cardia GC dataset using spatial Poisson regression model.

Parameter	Cardia GC		Non-cardia GC	
	IRR	95% Credible Interval (Lower, Upper)	IRR	95% Credible Interval (Lower, Upper)
**SESI**	0.859	(0.780, 0.947)	0.898	(0.812, 0.995)
**Immigrant**	0.994	(0.983, 1.003)	1.002	(0.990, 1.014)
**Indigenous**	0.986	(0.978, 0.994)	1.000	(0.990, 1.007)

From Table 2, it is evident that the covariates did not significantly predict the risk of GC for the overall GC dataset and the sex-stratified GC dataset. Table 3 presents the results for the topographical sub-site partitioning into CGC and NCGC. Unlike the overall result, SESI was a significant factor associated with CGC. A unit increase in SESI decreased the risk of CGC by 14%, and the risk of NCGC by 10%. 1% increase in regional Indigenous population proportion reduced the risk of CGC by 1.4% which was marginally significant, while the risk of NCGC among the Indigenous was not significant.

Further stratification of CGC by sex, as shown in Table 4, revealed no significant impact of SESI, immigrant and Indigenous variables on the risk of CGC for the men population. A different result for the female population was observed, where a unit increase in district SES score decreased the risk of CGC among women by a significant 26.2%, and the risk of CGC among Indigenous women population was reduced by 1.9% with 1% increase in regional Indigenous population proportion. The result of the non-cardia GC sex-stratified spatial model displayed in Table 5 showed no significant impact of the covariates on the risk of NCGC.

**Table 4:** Incidence Risk Ratio (IRR) and 95% credible interval for cardia GC dataset stratified by sex using spatial Poisson regression model.

Parameter	Male		Female	
	IRR	95% Credible Interval (Lower, Upper)	IRR	95% Credible Interval (Lower, Upper)
**SESI**	0.930	(0.827, 1.012)	0.738	(0.618, 0.879)
**Immigrant**	1.003	(0.986, 1.006)	0.994	(0.979, 1.009)
**Indigenous**	1.010	(0.982, 1.049)	0.981	(0.966, 0.996)

**Table 5:** Incidence Risk Ratio (IRR) and 95% credible interval for Non-cardia GC dataset stratified by sex using spatial Poisson regression model.

Parameter	Male		Female	
	IRR	95% Credible Interval (Lower, Upper)	IRR	95% Credible Interval (Lower, Upper)
**SESI**	0.910	(0.811, 1.022)	0.922	(0.819, 1.040)
**Immigrant**	1.010	(0.997, 1.022)	1.001	(0.987, 1.014)
**Indigenous**	1.001	(0.992, 1.010)	1.004	(0.995, 1.013)

**Fig 7:**
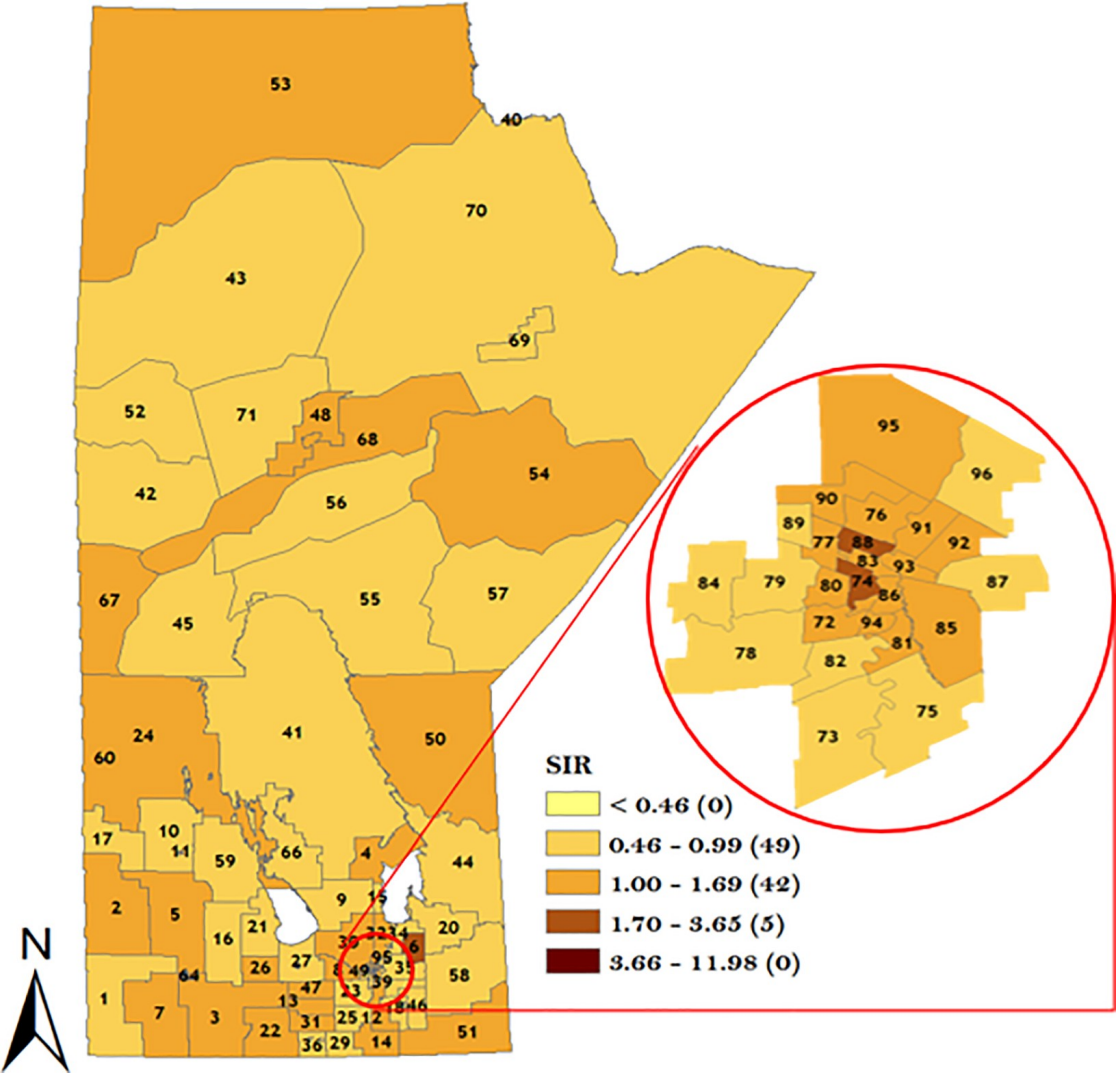
Map of standardized IRR of overall GC in Manitoba men using spatial Poisson regression model. Numbers in the map are the district labels from 1 to 96. Number of districts in each risk category is shown in parenthesis in the legends.

**Fig 8:**
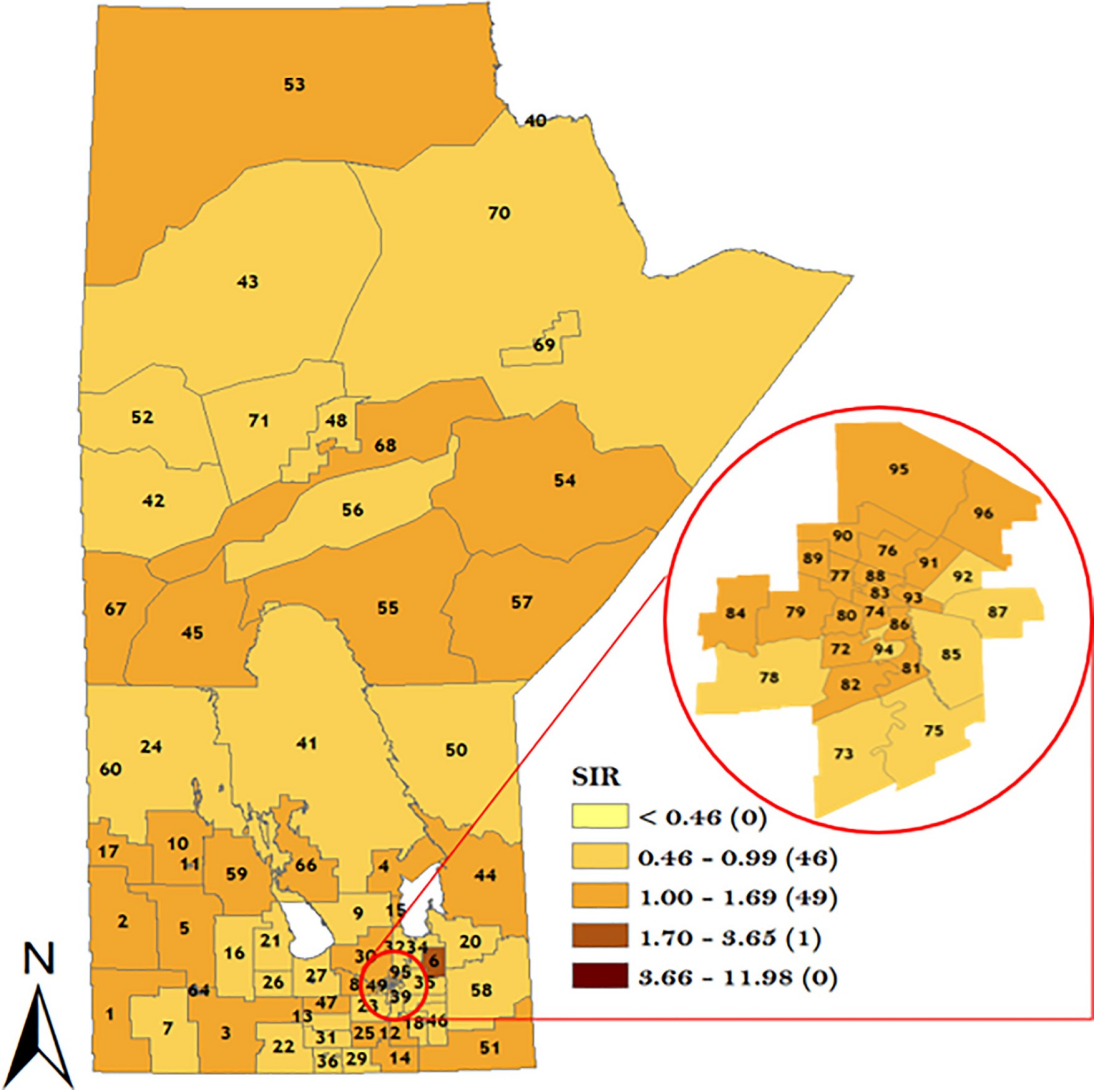
Map of standardized IRR of overall GC in Manitoba women using spatial Poisson regression model. Numbers in the map are the district labels from 1 to 96. Number of districts in each risk category is shown in parenthesis in the legends.

**Fig 9:**
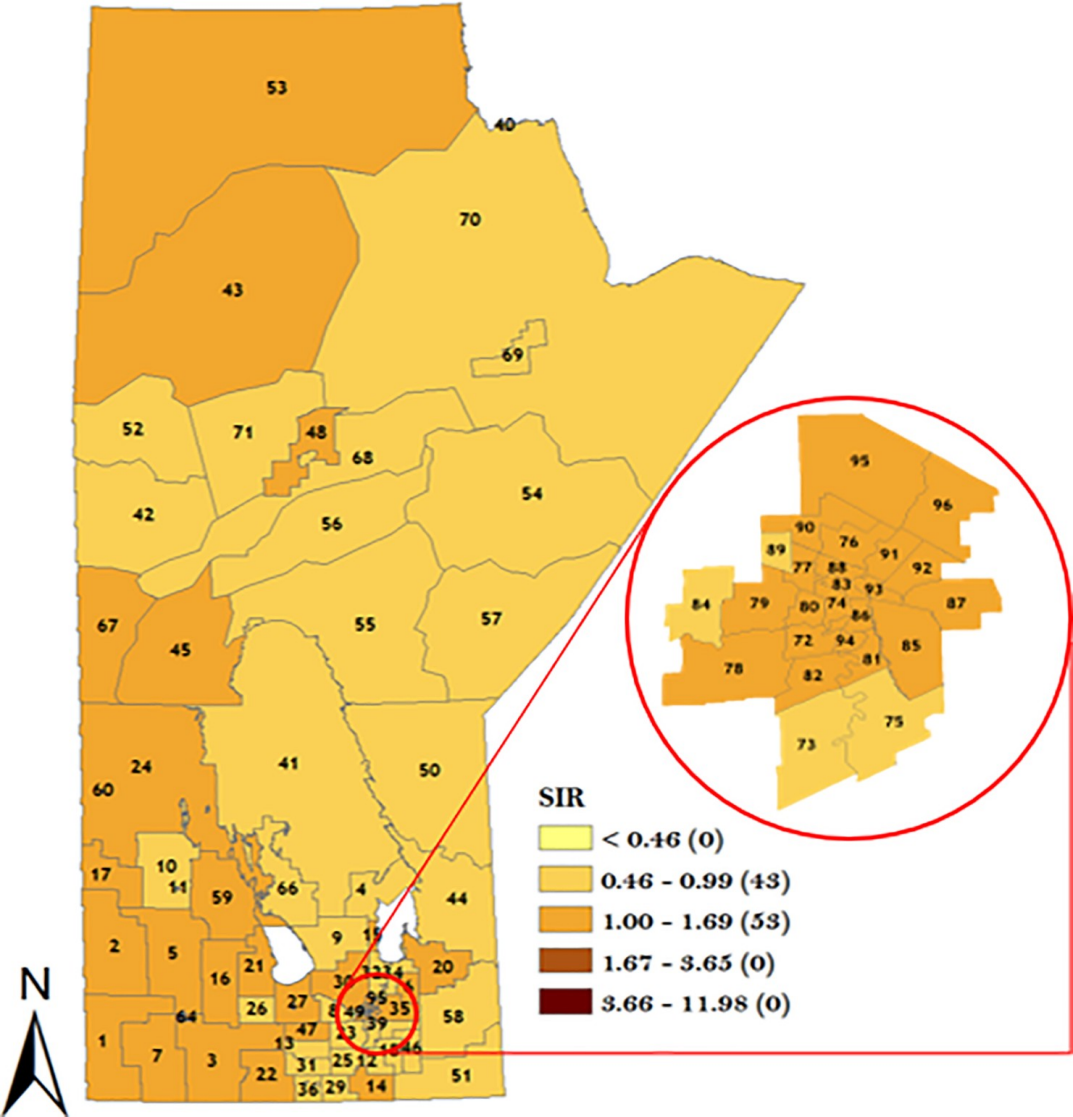
Map of standardized IRR of overall CGC in Manitoba using Poisson regression model. Numbers in the map are the district labels from 1 to 96. Number of districts in each risk category is shown in parenthesis in the legends.

**Fig 10:**
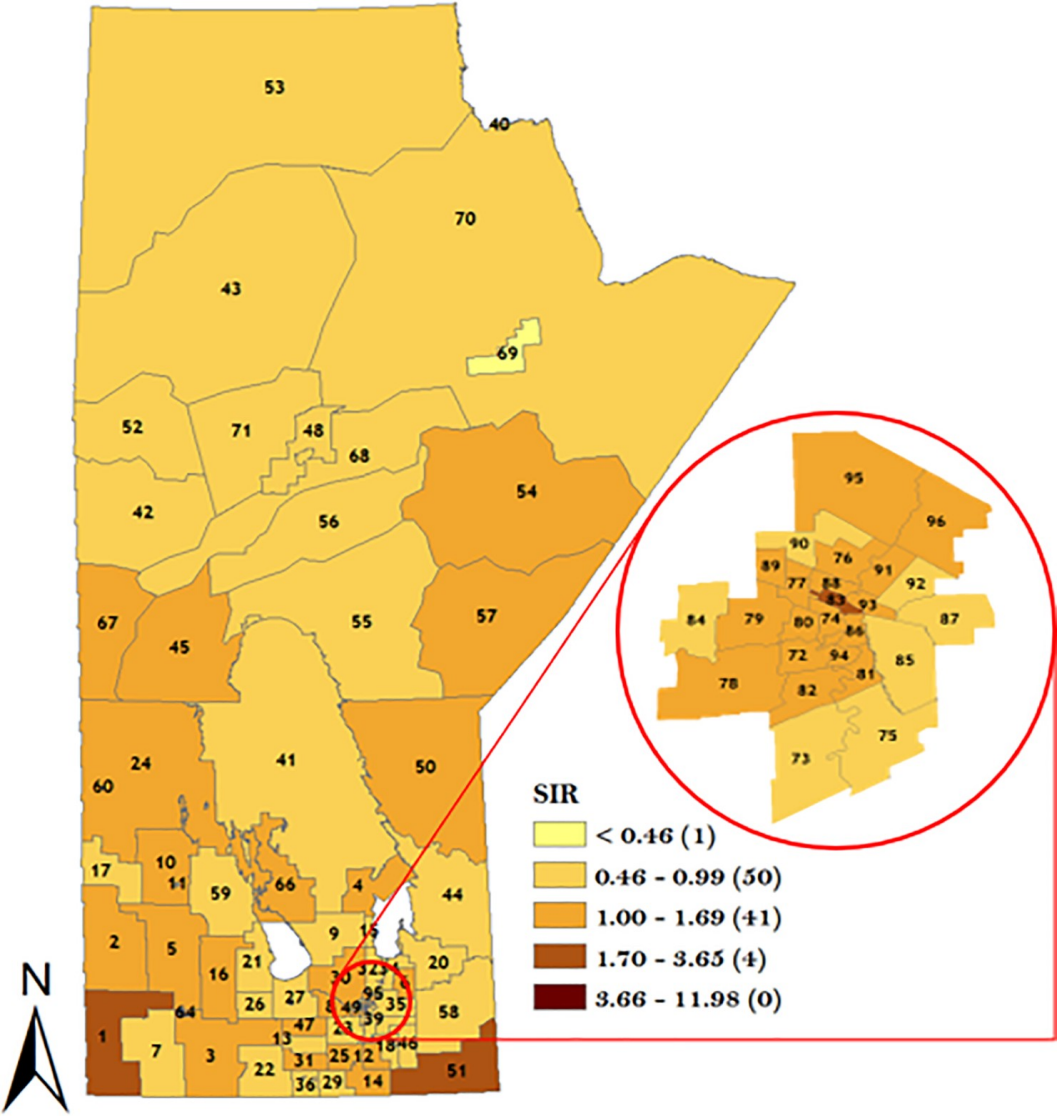
Map of standardized IRR of overall NCGC in Manitoba using Poisson regression model. Numbers in the map are the district labels from 1 to 96. Number of districts in each risk category is shown in parenthesis in the legends.

In order to understand the district-specific risk of GC stratified by sex and topographical sub-site, the smoothed spatial random effect from the model was plotted in Figs 7–10. The result of the spatial variation of GC stratified by sex for the male population is displayed in Fig 7 which identified 22 districts with GC incidence risk ratio greater than the rest of the population. Note that 7 RHADs out of the 22 RHADs (i.e., 32%) had a high GC incidence risk ratio relative to the rest of the population which were located in WRHA. Five districts (Winnipeg Churchill in the northern RHA, Brandon East end and Dauphin in Prairie Mountain RHA, and downtown East & Point Douglas North in Winnipeg RHA) had the highest risk ratios (between 1.70 and 3.65).

For the women GC sub-population, a total of 16 RHADs were identified with a high GC incidence risk ratio relative to the rest of the population. Note that 10 out of the 16 RHADs (i.e., 63%) were located in WRHA. One RHAD (district 06 in Interlake RHA) was identified with the highest GC incidence risk ratio as shown in Fig 8. The result of CGC spatial variation in Fig 9 identified 11 RHADs with a high CGC incidence risk ratio relative to the rest of the population. Note that 6 out of the 11 RHADs (i.e., 55%) were located in WRHA. The result of the NCGC in Fig 10 identified 27 RHADs with a higher risk of NCGC relative to the rest of the population. Note that 11 out of the 27 RHADs (i.e., 41%) with a high NCGC incidence risk ratio was located in WRHA. Similar to CGC, 4 RHADs (Souris River in Prairie Mountain RHA, Winnipeg Churchill in northern RHA, Rural East in Southern Health RHA, and Point Douglas South in Winnipeg RHA) were identified with the highest NCGC incidence risk ratios.

**Fig 11:**
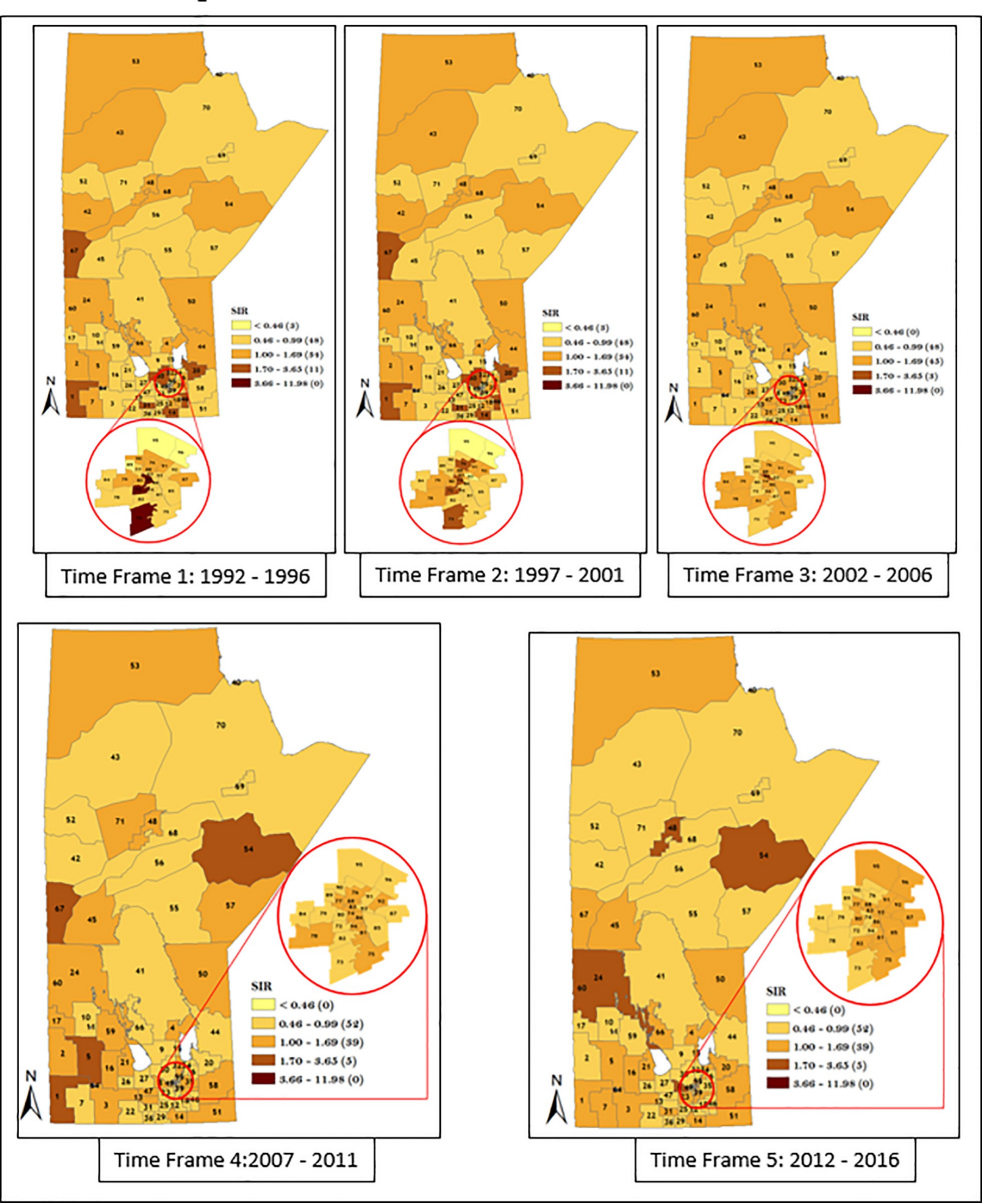
Maps of district-specific risk of overall men GC for the 96 RHADs in Manitoba for five time periods using spatio-temporal Poisson regression model. Numbers in the map are the district labels from 1 to 96. Number of districts in each risk category is shown in parenthesis in the legends.

**Fig 12:**
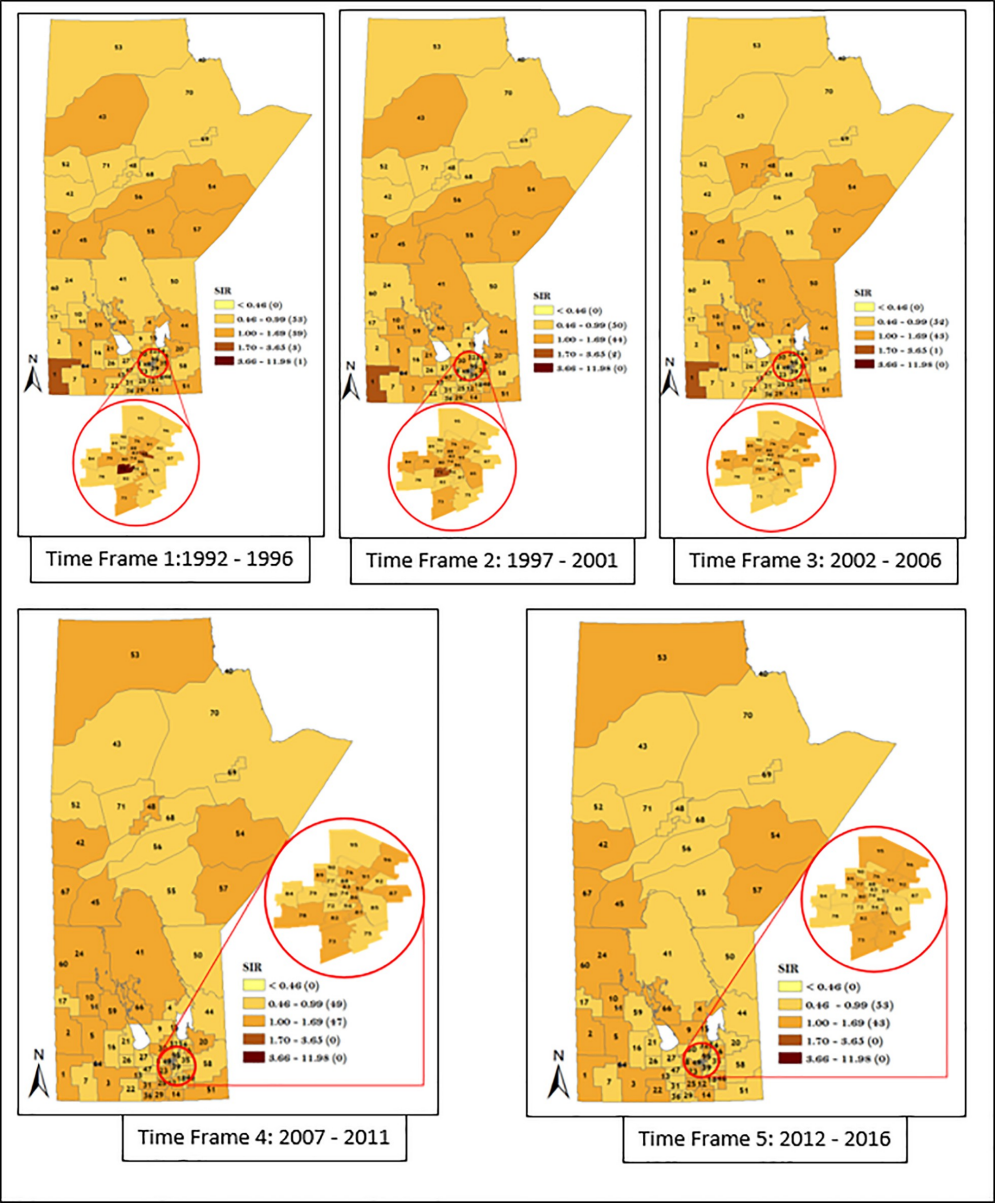
Maps of district-specific risk of overall women GC for 96 RHADs in Manitoba for five time periods, using spatio-temporal Poisson regression model. Numbers in the map are the district labels from 1 to 96. Number of districts in each risk category is shown in parenthesis in the legends.

**Fig 13:**
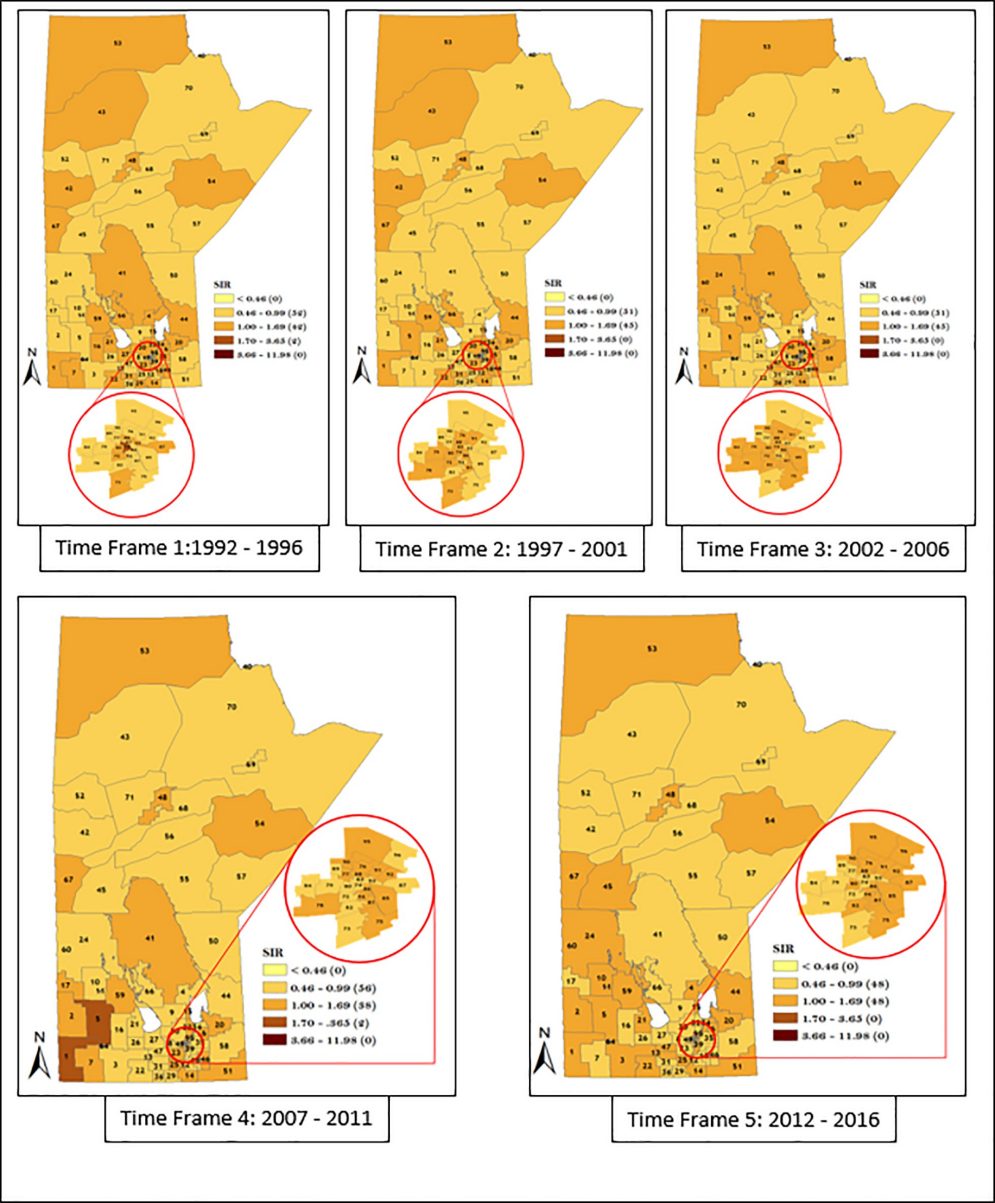
Maps of district-specific risk of CGC for 96 RHADs in Manitoba for five time periods using spatio-temporal Poisson regression model. Numbers in the map are the district labels from 1 to 96. Number of districts in each risk category is shown in parenthesis in the legends.

**Fig 14:**
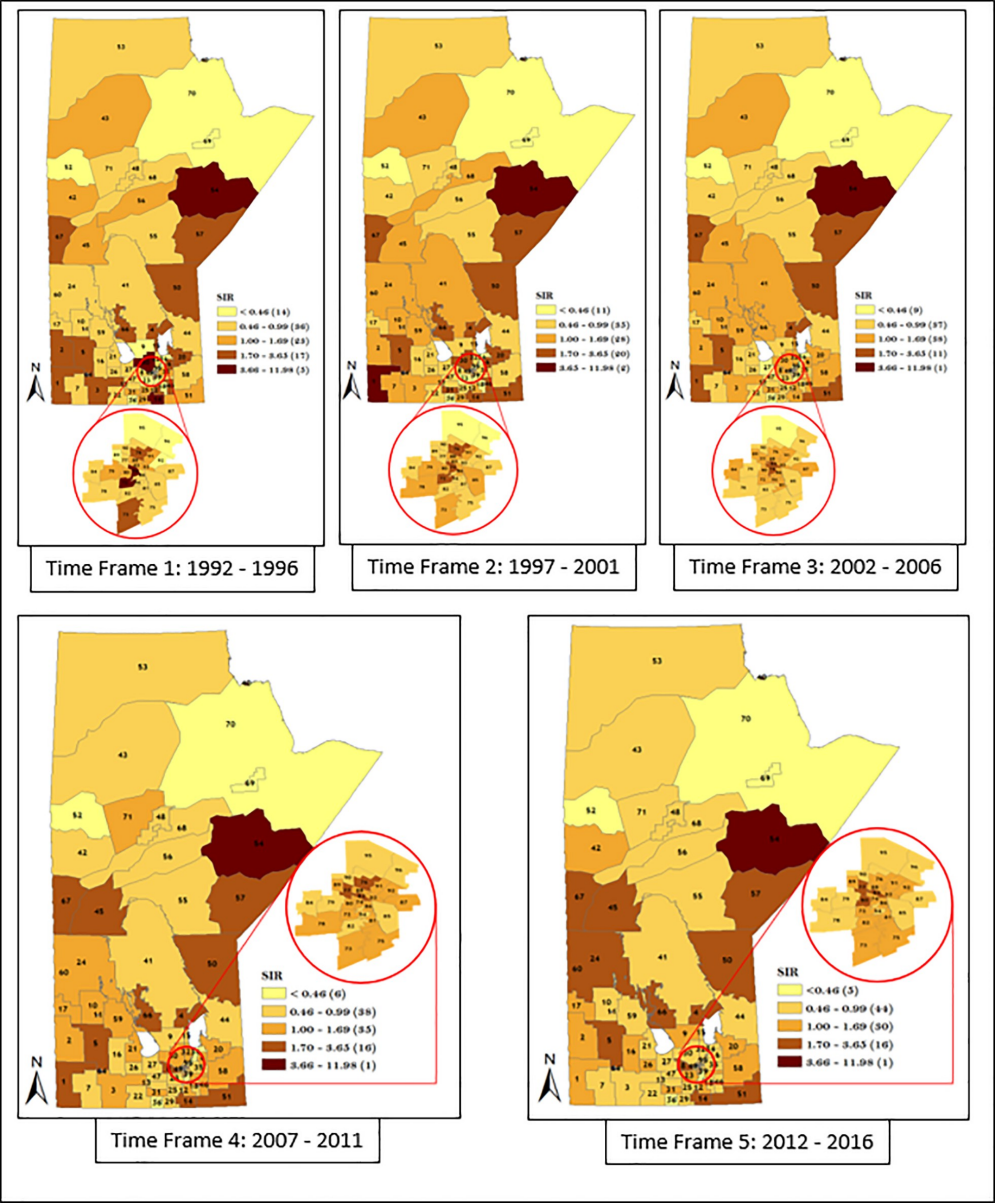
Maps of district-specific risk of NCGC for 96 RHADs in Manitoba for five time periods using spatio-temporal Poisson regression model. Numbers in the map are the district labels from 1 to 96. Number of districts in each risk category is shown in parenthesis in the legends.

Similarly, we also considered district-specific risk over time for each data strata, by mapping the spatio-temporal Poisson regression model (Figs 11–14). The results shown in Figs 11–14 revealed the presence of variation in risk of GC, CGC, and NCGC over time for each district. This is evident based on the variation in color gradient across the RHADs over time. More explanations about time trends of GC, CGC, and NCGC will be provided in the Discussion section.

## Discussion

In general, using the ecological regression model on the stratified dataset of GC, a significant association between SESI and CGC, and a marginally significant association between Indigenous population and CGC was observed, where both covariates decreased the risk of CGC. This result is partly supported by literature as some studies also suggest a decrease in the risk of GC among both sexes in Indigenous populations in other parts of the world [32]. Despite this, one might have predicted that Indigenous population proportion would be associated with a higher GC risk due to the fact that many of the Indigenous people in the study region have lower socioeconomic status and live in more remote regions, which could impede their access to good healthcare. We also observed a marginally significant association between SESI and NCGC.

A closer look at the district-specific risk identified five districts exhibiting high risk across all data sub-groups. These districts include Little Saskatchewan and Brandon East End in Prairie Mountain RHA and Point Douglas North, Downtown East, and St. Boniface West in WRHA. These districts were also associated with high percentage of no post-secondary school education (35.7% - 48.8%), unemployment (6.4% - 11.1%), a low income ($21,841 - $43, 648), and moderate proportion of immigrants (27.3%).

The spatio-temporal result presented in Figs 11–14 demonstrated apparent changes in the risk of GC across the RHADs over time. The study of the variation in risk of GC for each RHAD overtime gave us the opportunity to investigate regions with decreasing risk, increasing risk, and steady risk over time. Our findings support the fact that needs in respect to GC eradication across the province are not uniform. Therefore, policies regarding resource allocation for the detection and treatment of GC should be based on level of GC risk.

Until now, the epidemiological study of GC both in space and time in Manitoba was not well documented. Hence, this study was the first of its kind to examine the geographical variation of GC in Manitoba. This study offers a detailed glimpse into spatial epidemiology of GC in Manitoba. It will enable public health agencies (especially those with a keen interest in cancer surveillance) such as CancerCare Manitoba and Manitoba Health to have a clear picture of the spatial variation of GC in Manitoba. This study will then help policy makers determine resource allocation and plan for possible prevention/intervention. In addition to using the findings in this study for a logical resource allocation program, it will also serve as a GC diseases surveillance and monitoring tool for interested organizations.

### Strengths and limitations

One of the main strengths of this study is that it is based on population data and therefore not subject to selection bias. The spatial and spatio-temporal methods used in this study leveraged on districts neighborhood to obtain a reliable risk estimate. The inclusion of time random effect also helped us to acknowledge and account for time effect, thereby avoiding over-estimation. The time effect also helped us investigate the trend of the GC over time, which is a desired tool in disease trend surveillance. The main advantage of using spatio-temporal regression model is to capture all complex variations (risk factors and spatio-temporal variation) unlike the other models such as e.g. geographically weighted regression. One could also use cluster detection methods such as SaTScan [33] which is a simulation-base method to identify clusters with high risk without properly assuming necessary assumptions on the nature of outcome. Also, the cluster detection methods are not able to simultaneously identify possible risk factors of the disease (e.g., GC) unlike our sophisticated spatio-temporal regression model. However, this study is limited by the fact that we were unable to adjust for several other factors such as smoking, obesity, lifestyle, Helicobacter pylori, and food, which are important risk factors associated with the etiology of GC. Some of factors used in this work such as Indigenous and Immigrant population may be used as proxy for covariates such as lifestyle and food.

## Conclusions

In conclusion, our study has demonstrated that the risk of GC varied across the province of Manitoba.

Not only was the incidence risk of GC found to differ across small regions within the province, several regions exhibited different progressive risk (decreasing, increasing or steady) over time. While the overall incidence risk of GC at the provincial level was steady over time, this was not true for all RHADs. More studies to investigate the driving force behind the progressive increase in risk of GC for the identified regions are highly recommended. This will further enable policy makers to address the issue more efficiently as each district may require a different approach to eradicating GC risk.

Finally, we demonstrated the use of spatial and spatio-temporal regression model as a robust model for investigating variation in space and time. The model leverages on both regional and temporal dependency to arrive at a more reliable estimate. This modeling approach is highly recommended in health study where the dataset is collected over space and time.
